# The Potential Role of Wearable Inertial Sensors in Laboring Women with Walking Epidural Analgesia

**DOI:** 10.3390/s24061904

**Published:** 2024-03-16

**Authors:** Mikhail Dziadzko, Adrien Péneaud, Lionel Bouvet, Thomas Robert, Laetitia Fradet, David Desseauve

**Affiliations:** 1 Département d’Anesthésie-Réanimation, Hôpital de la Croix-Rousse, Hospices Civils de Lyon, Université Claude Bernard, F-69004 Lyon, France; 2 U1290 RESHAPE, INSERM, Université Claude Bernard Lyon 1, F-69008 Lyon, France; 3 Laboratoire de Biomécanique et Mécanique des Chocs UMR_T 9406, Univ Eiffel, Univ Lyon 1, F-69622 Lyon, France; 4 Equipe Robotique, Biomécanique, Sport, Santé, Institut PPRIME, UPR3346 CNRS, Université de Poitiers ENSMA, 11, Boulevard Marie et Pierre Curie, Site du Futuroscope, CEDEX 9, F-86073 Poitiers, France; 5 Département d’Anesthésie-Réanimation, Hôpital Femme Mère Enfant, Hospices Civils de Lyon, Université Claude Bernard, F-69500 Bron, France; 6 Obstetric Research Lab, Department of Women-Mother-Child, Lausanne University Hospital (CHUV), 1005 Lausanne, Switzerland; 7 Child Couple University Hospital, University of Grenoble, F-38700 Grenoble, France

**Keywords:** biomechanics, gait analysis, labor, prediction, sensors, walking epidural

## Abstract

There is a growing interest in wearable inertial sensors to monitor and analyze the movements of pregnant women. The noninvasive and discrete nature of these sensors, integrated into devices accumulating large datasets, offers a unique opportunity to study the dynamic changes in movement patterns during the rapid physical transformations induced by pregnancy. However, the final cut of the third trimester of pregnancy, particularly the first stage of labor up to delivery, remains underexplored. The growing popularity of “walking epidural”, a neuraxial analgesia method allowing motor function preservation, ambulation, and free movement throughout labor and during delivery, opens new opportunities to study the biomechanics of labor using inertial sensors. Critical research gaps exist in parturient fall prediction and detection during walking epidural and understanding pain dynamics during labor, particularly in the presence of pelvic girdle pain. The analysis of fetal descent, upright positions, and their relationship with dynamic pelvic movements facilitated by walking during labor is another area where inertial sensors can play an interesting role. Moreover, as contemporary obstetrics advocate for less restricted or non-restricted movements during labor, the role of inertial sensors in objectively measuring the quantity and quality of women’s movements becomes increasingly important. This includes studying the impact of epidural analgesia on maternal mobility, walking patterns, and associated obstetrical outcomes. In this paper, the potential use of wearable inertial sensors for gait analysis in the first stage of labor is discussed.

## 1. Introduction

Wearable inertial sensors include accelerometers and gyroscopic measurements. Accelerometers assess the rate of change in linear velocity, and gyroscopes measure the angular velocity. These sensors are thus typically combined into inertial measurement units (IMUs) and are found in fitness trackers and smartwatches for monitoring a user’s physical activity, balance, and real-time orientation. By processing data from these sensors, wearable devices can provide valuable insights into user movements, enabling applications ranging from sport movement analysis to augmented reality experiences or medical monitoring [1].

Various data-processing technologies have been proposed for these sensors. Acceleration and angular velocity can be combined to obtain orientation and position information through data fusion filters such as Kalman filters [2]. Inertial-based motion capture systems are increasingly considered a viable alternative to traditional optoelectronic-based human motion capture [3]. These sensors also enable the use of feature extraction techniques such as Fourier transform or wavelet analysis, and machine learning algorithms can also be employed for activity recognition or anomaly detection in motion [1,4].

There is a growing interest in the use of inertial sensor data in pregnant women [5,6,7]. This interest comes from the noninvasive and discrete nature of sensors, which may be integrated into devices able to accumulate large amounts of longitudinal data, thus enabling movement analysis during pregnancy.

Pregnancy is characterized by profound anatomical and physiological changes that affect maternal biomechanics and kinematics [8,9]. Standard antenatal maternal and fetal monitoring during pregnancy is based on routine care visits and laboratory tests. Its objectives include early detection of fetal abnormalities, assessment of fetal well-being, monitoring maternal health, and identifying risk factors to facilitate timely interventions, thereby optimizing pregnancy outcomes. However, the discontinuous schedule of such visits may not accurately identify all potentially threatening changes that may occur between appointments, particularly those linked to morphological and biomechanical changes such as altered spinal curvature, body balance, and gait.

The primary concern resulting from these changes is the increased risk of falls, which escalates throughout pregnancy [10]. Research indicates that up to 80% of women experience falls during the third trimester, with a higher prevalence observed in those exhibiting altered dynamic postural stability [11], associated with changes in gait and posture [12].

Gait modulation during pregnancy is influenced more significantly by pelvic girdle pain than the pregnancy itself. Women experiencing such pain tend to demonstrate slower and more rigid walking patterns compared to asymptomatic pregnant women, likely due to alterations in load dynamics. Moreover, alterations in the anti-phase coordination between the pelvis and thorax are particularly pronounced in those experiencing pregnancy-related pelvic girdle pain [13].

These variations in gait and posture can be effectively captured by a multiple-point or single-point wearable inertial measurement unit [14], translating into pattern analysis and fall prediction systems.

Anticipated and expected biomechanical adjustments during the first stage of labor include the following:

- A forward shift in the center of gravity, likely induced by increased lumbar lordosis and pronounced anterior pelvic tilt, and due to fetal descent.
- A broader stance and augmented pelvic joint mobility resulting from pelvic ligament laxity.
- Potentially wider gait patterns influenced by an expanded pelvic outlet.
- Adaptive pelvic motions to accommodate fetal descent.
- Pain-driven alterations in lower extremity kinematics characterized by abbreviated step lengths and reconfigured weight distribution [15,16,17,18].

**Figure 1:**
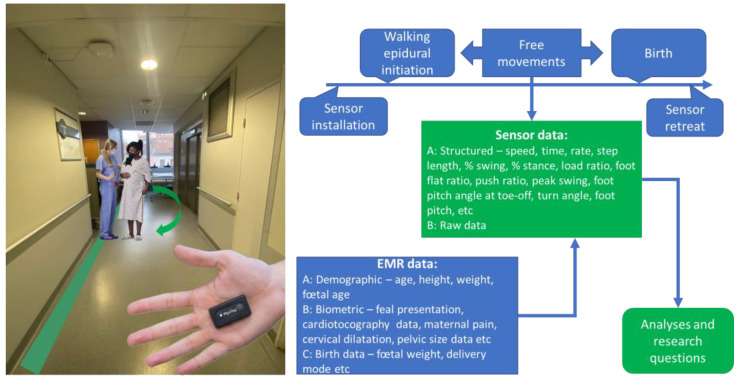
The conceptual integration of wearable sensors into obstetric biomechanical studies. The illustration shows the size of a wearable inertial sensor (Pysilog 5^®^; GateUp here). EMR—electronic medical record data. The data listed are not exhaustive.

Throughout the first stage of labor, controlled pain conditions prompt biomechanical gait adaptations, reflecting the evolving anatomical and physiological demands. Factors such as cervical dilation, uterine contractions, and changes in pelvic geometry and fetal positioning collectively influence posture, pelvic stability, and lower extremity kinematics (Figure 1).

The contemporary obstetrics paradigm advocates for less restricted or non-restricted movements during labor, underlying the importance of maintaining maternal biomechanics as close to physiological as possible [19,20,21]. Understanding obstetrical biomechanics within the context of physiological childbirth requires a comprehensive exploration of factors influenced by maternal and fetal dynamics [22]. While knowledge of birth biomechanics in the traditional lithotomy position, commonly employed as the standard delivery position in hospital settings [23], is essential, it is insufficient for analyzing and managing fetal progression and associated outcomes in alternative birthing postures such as squatting or hands-and-knees. These alternative positions portray childbirth as a physical achievement, underscoring the importance of selecting the most effective positions [22,24]. Epidemiological data suggest numerous benefits associated with vertical birthing positions, yet further research is needed to investigate the role of verticalization during the first stage of labor [25].

More complex interactions during the first stage of labor may appear during upright positions and walking. Theoretically, the force of gravity should aid in the descent of the fetal head and in the optimal alignment of the fetus within the maternal pelvis, potentially facilitating the opening of the pelvic outlet. Maternal gait involves pelvic movements, which can contribute to the dynamic changes in the pelvic anatomy during labor.

The dynamic nature of labor, individual anatomical variabilities, limited imaging options, safety concerns, interaction with medical interventions, and, finally, ethical considerations, including privacy and dignity, pose challenges to the systematic exploration and study of childbirth biomechanics during labor during unrestricted maternal movements and alternative birthing postures.

Recent advances in pain control, particularly during labor, allow pregnant women to ambulate during the first stage of labor until complete cervical dilatation. This technique, known as “walking epidural”, evolved from low-concentration patient-controlled epidural analgesia, enabling free movement and walking during the first stage of labor [26,27].

Multimodal pain control with preserved motor function and, most importantly, less restricted or non-restricted movements during labor have been studied using traditional outcome measurements such as pain level, opioid consumption, duration of birth phases, maternal satisfaction, neonatal outcomes, and many others [21,28,29,30]. None of these studies assess the quality and quantity of women’s movements during labor, and the published results, which at time conflict, are not adjusted for movement quantity. Interactions between advanced obstetrical analgesia methods (such as low-dose epidural), the ability to walk, upright position, quality and quantity of gait, and the biomechanics of labor, particularly during the first stage of labor, are rarely investigated. Remarkably, the nuances of gait during ambulation with low-concentration epidural labor analgesia remain unexplored.

## 2. Walking Epidural, a Modern Anesthetic Trend in Obstetrics, Beckoning Patients and Caregivers

Walking epidural analgesia is considered a significant advancement in pain management during labor when compared to traditional epidural techniques [21]. Achieving a delicate balance between pain relief and preserving muscle strength and sensation enables adequate analgesia while allowing for greater maternal mobility [26]. This mobility empowers parturients to remain ambulatory, change positions, and even walk during labor, enhancing their childbirth experience and promoting optimal labor progress.

Furthermore, the reduced impact on motor function associated with walking epidurals decreases the likelihood of assisted interventions, such as forceps or vacuum delivery, which can become necessary due to decreased pushing ability with traditional epidurals. Engaging in upright positions facilitated by walking epidurals can enhance the efficiency of contractions and potentially accelerate labor.

The average duration of the active first stage of labor, from the onset of contractions to full cervical dilation, varies from 6 to 17 h for nulliparous women and is typically shorter for multiparous women [31]. Following the initiation of low-concentration epidural analgesia, the average reported duration of ambulation is 25 to 60 min, or at least 5 min per 1 h of the first stage [32,33].

The standard protocol for walking epidural includes a series of hemodynamic and neurological clinical assessments: orthostatic hypotension and bradycardia detection, evaluation of muscular weakness, and assessment of proprioception alterations to mitigate the risk of falls or cardiovascular repercussions on the fetus. The absence of these signs is mandatory for permitting maternal ambulation. During ambulation, various safety monitoring approaches are employed, such as intermittent or continuous wireless fetal monitoring, intermittent point-of-care or wearable wireless hemodynamic monitoring (blood pressure and heart rate), and direct observation. In cases of repeated bolus administration for insufficient analgesia, immobilization of the parturient in bed is required to investigate the situation; clinical tests are repeated if ambulation resumes due to the potential risk of motor block or orthostatic hypotension.

Differential nerve block induced by low-concentration local anesthetics alleviates the painful sensations associated with labor progression. However, breakthrough pain may occur during ambulation in certain circumstances, including rapid changes in pelvic geometry, rapid cervical dilatation, epidural catheter displacement, fetal dystocia, and uterine rupture (in the context of a previous cesarean section). The evolution of pain may be gradual over minutes, influencing the parturient’s gait pattern. Conversely, pelvic dilatation resulting from the fetus’s progression toward the birth canal may alter gait biomechanics.

## 3. Research Gaps Addressable with Inertial Sensors

### 3.1. Prediction and Detection of Falls during Walking Epidural

To the best of our knowledge, there are no studies reporting the use of inertial wearable sensors for the prediction and detection of falls during walking epidural administration in pregnant women. A longitudinal study of pregnant gait biomechanics demonstrates the increased falling risk during the 3rd trimester of pregnancy associated with changes in lower extremity kinematics and foot pressure alterations [34]. The assessment of gait velocity and heel strike angle using mobile inertial sensors has been shown to be essential kinematic indicators in a fall risk screening test [35]. Biomechanical video-based analysis shows that to ensure safe motion, pregnant women should not initiate gait until reaching a stable standing position after rising [36]. However, no valid data currently exist regarding the conditions of ambulation with low-dose epidural walking analgesia. Such analgesia may induce a proprioceptive nerve block that is likely present but clinically undetectable. This could potentially contribute to maternal instability during vertical positions, thereby increasing the risk of falls. Time series data collected from inertial sensors on ambulating women with epidural analgesia during the first stage of labor hold promise in addressing these questions: does low-dose epidural analgesia alter gait patterns, increase the risk of falling, and can such falls be predicted?

### 3.2. Pain Detection

Pelvic girdle pain, exacerbated by the physiological changes due to pregnancy and labor, is a prevalent concern among pregnant women [17,37]. This pain may become excruciating during labor. Walking epidural analgesia offers a unique opportunity to study pain dynamics while maintaining mobility. Low-concentration epidural analgesia provides selective blocking of non- and less-myelinated nerve fibers [38]. However, intense nociceptive stimulation resulting from mechanical constraints (such as fetal position changes or dystocia leading to maternal compressive ischemia of internal organs, compression of nerve structures, rapid pelvic dilatation, or even uterine rupture) will produce intense pain sensations despite the presence of a partial nerve block. These conditions are particularly noteworthy in obstetrics due to their association with unfavorable maternal and fetal outcomes.

The particular case of dystocia, a complex obstetric challenge associated with increased rates of maternal interventions and adverse neonatal outcomes, continues to be a subject of ongoing research. Early prediction of dystocia enables timely interventions, thereby improving maternal and neonatal survival rates. Recent animal studies utilizing inertial sensors to detect and predict parturition and uterine diseases demonstrate the feasibility of incorporating movement analysis in such contexts [39]. No human studies investigating the relationship between data from inertial sensors and modification of movement patterns in conditions of breakthrough pelvic girdle pain were found. Pain-controlled adaptations, including reduced stride length, decreased walking speed, altered pelvic tilt, and increased lumbar lordosis, are aimed at minimizing discomfort and strain on sensitive pelvic structures. Recognizing these modifications with the help of inertial sensors offers a new level of safety monitoring and situational awareness during labor.

### 3.3. Fetal Descent

Upright positions, such as standing, sitting, kneeling, squatting, and walking, are thought to promote the progression of the first stage of labor, although reported observations are conflicting [22,25,40]. Gravity force and dynamic pelvic movements, particularly during walking, are believed to contribute to better pelvic inlet, fetal alignment, rotation, and descent. In the context of epidural analgesia, pelvic girdle pain may be absent, shifting the focus of gait and posture changes to alterations in the gravity center and the alignment of the spine, pelvis, and hips. These alterations have the potential to generate distinct patterns that are probably measurable and analyzable using inertial sensors, and additional data such as pelvic perinatal measurements (pelvimetry) may be required. No communications or proof-of-concept studies are available to date.

### 3.4. Promoting Approaches to Limit Intervention during Labor and Birth

The degree of medicalization of labor varies significantly across countries, with a discernible trend toward limiting medical interventions during childbirth [41]. This trend, however, contradicts the notably high rates of epidural analgesia, particularly in France, where over 80% of women opt for neuraxial analgesia as a primary pain management method [29]. Despite conflicting evidence and country-specific variations, existing research demonstrates an association between the occurrence of postpartum depression and deliveries involving epidural anesthesia [42,43,44]. Among the numerous sources of dissatisfaction reported by women with satisfactory pain control are restrictions linked to limitations on free movements, the use of urinary catheters, and the reluctance of medical staff to permit food intake during labor [45,46,47]. The well-established association between negative childbirth experiences and the subsequent risk of postpartum depression is further underscored by recent data suggesting that postpartum depression, along with cardiac diseases, constitutes a primary cause of maternal mortality within one year after childbirth [48,49].

While these conclusions are deductive and speculative, one may argue that commonly employed measurement instruments may lack specificity or sensitivity in deciphering the complex interplay of various perinatal factors influencing maternal satisfaction. An illustrative example is intrapartum fasting, a subject of frequent pro and con debates. Each side presents valid arguments rooted in security requirements, such as the avoidance and prevention of gastric content inhalation in the case of general anesthesia and the imperative need to address parturients’ requirements.

Decreased gastrointestinal mobility, varying up to gastroparesis, is prevalent during pregnancy, yet the literature on this topic is scarce [50]. Research on the effects of exercises on gastrointestinal transit [51] suggests that moderate physical activity, such as walking, during early labor may enhance gastric emptying.

The effect of labor analgesia on gastric emptying has been recently studied [52,53]. However, no data on the relationship between the quantity of any type of physical activity (free movements, walking) are available. Filling in this research gap is important because free walking with pain relief and stimulating gastric emptying may not only reduce the risk of maternal aspiration if general anesthesia is required but also contribute to a holistic approach to women’s comfort during labor. Wearable sensors provide readily available quantities of movements (steps) in upright positions, allowing for direct, measurable relationships with the gastric content and the rate of gastric emptying. This facilitates the design of high-quality randomized studies to fill in this research gap.

## 4. Challenges Associated with Using Wearable Inertial Sensors for Gait Analysis during the First Stage of Labor

Wearable inertial sensors are small, with sizes varying from extremely small ones to the size of a matchbox [54]. Different types are commercially available, and the availability of OEM parts allows for custom research-oriented sensor building. Sensor type, number (single or multiple), sensor placement (trunk or extremity), study aims, measurement dataset, and data treatment methods may vary depending on the research question [14]. As no studies in the context of maternal ambulation in labor exist, it is difficult to analyze the specific challenges associated with using wearable inertial sensors in such conditions. The most common problems may arise from the data quality (parasite readings, non-recognition of maternal activity) and quantity (limited time for ambulation) collected. Synchronization of sensor data and clinical events may present another challenge. Finally, the reluctance of parturients to participate in this research may present a classical obstacle.

Observational, large-scale longitudinal studies with a pilot design and the use of a big data analysis approach may help guide the initial research.

## 5. Conclusions

The integration of wearable inertial sensors in studies on laboring women represents a promising avenue for advancing our understanding of biomechanics during childbirth. Addressing the identified research gaps using inertial wearable sensors can contribute to fall prediction, studies on pain dynamics, monitoring fetal descent, and developing interventions targeting maternal satisfaction improvement, ultimately enhancing mothers’ and infants’ safety and well-being during the labor process.

The discrete nature of wearable sensors, their autonomy in measurement, and the capability to store and accumulate temporal series data offer undeniable advantages for translational research in obstetrics. Therefore, we encourage obstetrical teams to prioritize and promote observational research aimed at gathering data from inertial sensors in laboring women. This proactive approach will not only contribute to the refinement of existing knowledge but also foster innovation in maternal and infant care, ultimately improving the safety and well-being of mothers and infants during the labor process.
